# Supplementary material for: RNA-Binding Protein MEX3A Interacting with DVL3 Stabilizes Wnt/β-Catenin Signaling in Endometrial Carcinoma
Source: Int J Mol Sci. 2022 Dec 29;24(1):592. doi: 10.3390/ijms24010592 (PMC9820120; doi:10.3390/ijms24010592)
Supplement: Supplementary file 1 [file ijms-24-00592-s001.zip › ijms-2044138-supplementary.pdf]

Supplementary Materials

1.Supplementary Tables

Table S1. si-RNA sequences used in this study.

	sense（5'-3'）	antisense（5'-3'）
MEX3A(h)-si-1	GCAUCCAGCAGCAAACCAATT	UUGGUUUGCUGCUGGAUGCTT
MEX3A(h)-si-2	GCGGAGUGGACUCUGGCUUTT	AAGCCAGAGUCCACUCCGCTT
DVL3(h)-si-1	CAGGUAAACGAGAUCAACUUUTT	AAAGUUGAUCUCGUUUACCUGTT
DVL3(h)-si-2	GCGACCCAGCUAUAAGUUCUUTT	AAGAACUUAUAGCUGGGUCGCTT
MEX3A(h)-sh-1	GCGGAGTGGACTCTGGCTT	
MEX3A(h)-sh-2	GCTTTGGAGAACTAGGATGTT	
DVL3(h)-sh-1	GCATGCAACCCGGCTAAAT	
DVL3(h)-sh-2	GCAGAAGGTTTCTCGGATT	

Table S2. Sequences of primers used in this study.

Gene	Forword Sequence（5'-3'）	Reverse Sequence（5'-3'）
MEX3A	TGGAGAACTAGGATGTTTCGGG	GAGGCAGAGTTGATCGAGAGC
GAPDH	GGAGCGAGATCCCTCCAAAAT	GGCTGTTGTCATACTTCTCATGG
β-catenin	GGCTCTTGTGCGTACTGTCCTTC	GCTTCTTGGTGTCGGCTGGTC
c-Myc	CCTGGTGCTCCATGAGGAGAC	CAGACTCTGACCTTTTGCCAGG
Cyclin D1	TCTACACCGACAACTCCATCCG	TCTGGCATTTTGGAGAGGAAGTG
CD44	TGGCACCCGCTATGTCCAG	GTAGCAGGGATTCTGTCTG
E-cadherin	CGAGGCTACACGTTCACGG	GGGTGTCGAGGGAAAAATAGG
N-cadherin	TCAGGCGTCTGTAGAGGCTT	ATGCACATCCTTCGATAAGACTG
Vimentin	TCTAGGAGGAGATGCGG	GGTCAAGACGTGCCAGAGAC
Snail	TCGGAAGCCTAACTACAGCGA	AGATGAGCATTG-GCAGCGAG
Slug	CTGTGACAAGGAATATGTGAGC	CTAATGTGTCCTTGAAGCAACC

Table S3. Antibodies used in this study

Antibodies	Source
MEX3A	Abcam ab79046
β-catenin	ABclonal A0316
c-Myc	ABclonal A1309
Cyclin D1	ABclonal A0310
CD44	ABclonal A12410
E-cadherin	Servicebio GB11868

N-cadherin	Servicebio GB111009
Vimentin	ABclonal A19607
Snail	ABclonal A11794
Slug	ABclonal A13352
DVL3	Proteintech 13444-1-AP
anti-Flag	ABclonal AE005
HRP-anti-Rabit	Servicebio G1213
HRP-anti-Mouse	Servicebio G1214
GAPDH	Servicebio GB11002

2. *Supplementary Figures*

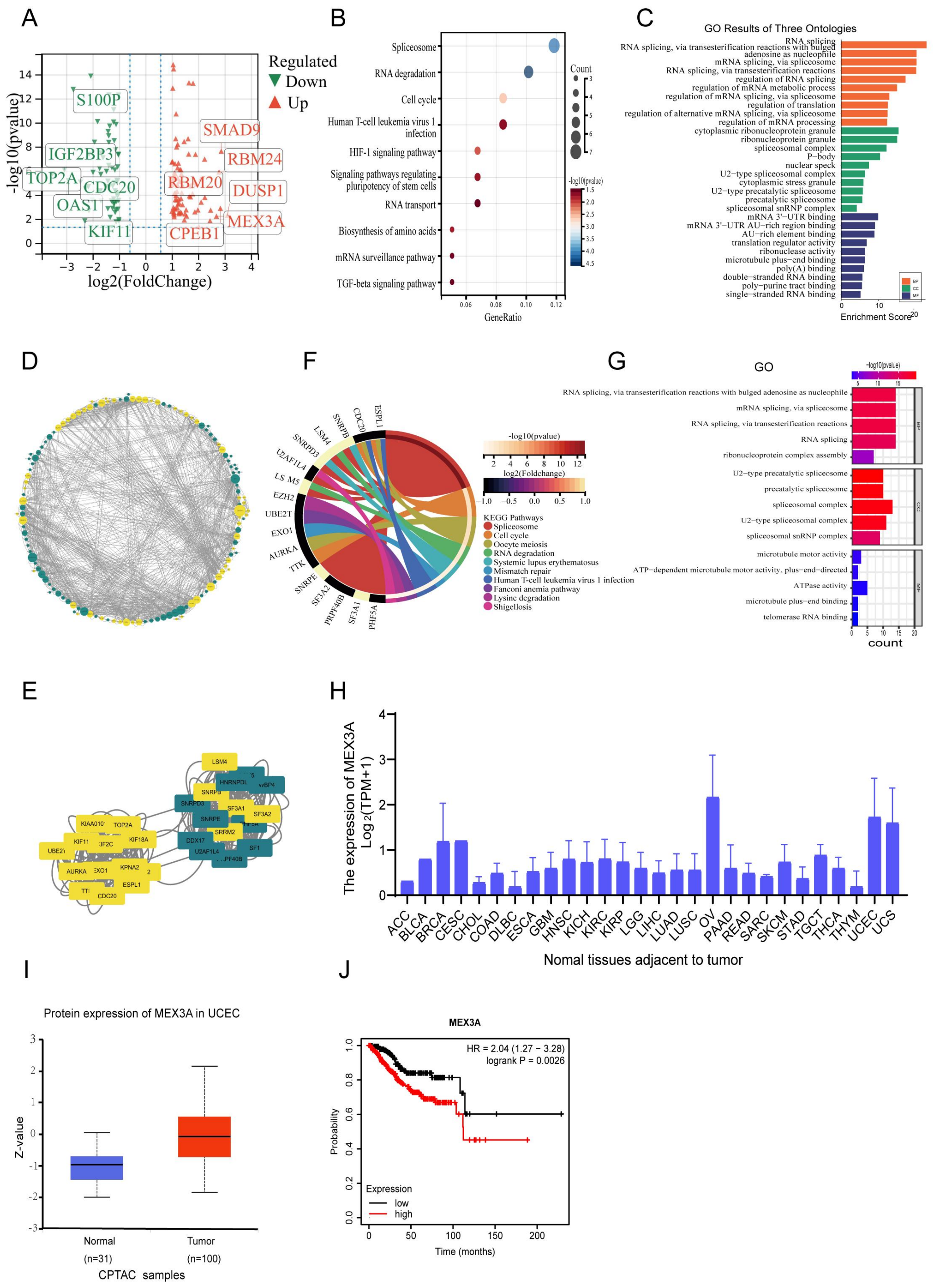

Supplementary Figure S1. Identification of differentially-expressed RBPs (DE RBPs) in EC. (A) Volcano plot displaying detailed information on the DE RBPs. (B–C) KEGG and GO analyses of the DE RBPs. (D) Protein – protein interaction (PPI) network of the identified DE RBPs. Blue indicates upregulated RBPs. Yellow indicates downregulated RBPs. (E) Key co-expressed modules of the PPI network. (F–G) KEGG and GO analyses of the key modules. (H) The MEX3A expression levels of 28 normal tissues adjacent to tumors. (I) Protein expression of MEX3A in EC and normal tissues based on CPTAC. (J) Kaplan – Meier analysis of relapse-free survival (RFS) related to the expression of MEX3A. *$P < 0.05$, **$P < 0.01$, ***$P < 0.001$.

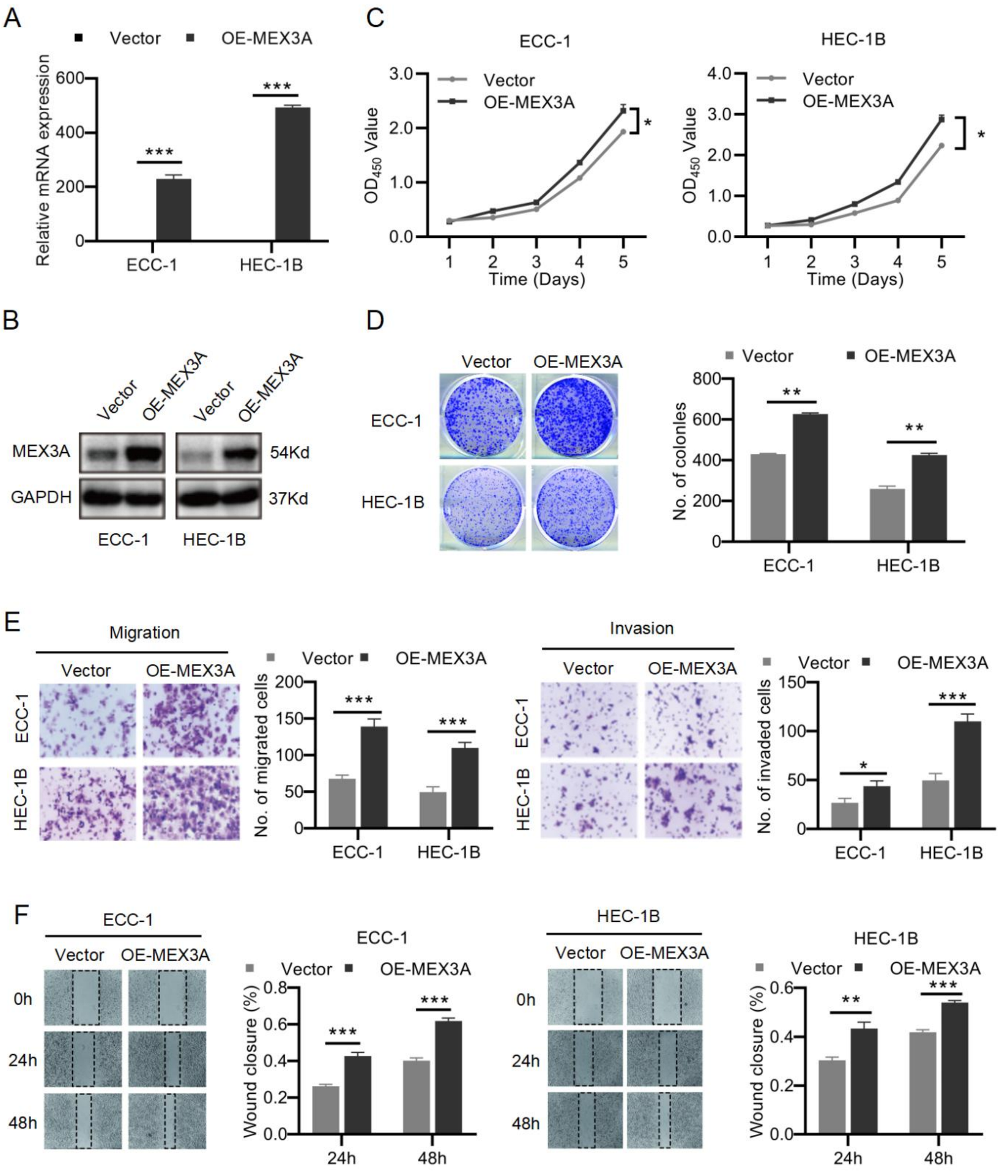

Supplementary Figure S2. MEX3A overexpression enhances the proliferation and metastasis of EC cells. (A−B) The efficacy of the overexpression MEX3A was detected by qRT-PCR and western blotting. (C–D) The CCK-8 and colony assays showed that MEX3A overexpression promoted the proliferation of ECC-1 and HEC-1B cells. (E–F) The transwell and wound healing assays analyzed the migration and invasion abilities of ECC-1 and HEC-1B cells after MEX3A overexpression. **P* < 0.05, ***P* < 0.01, ****P* < 0.001.

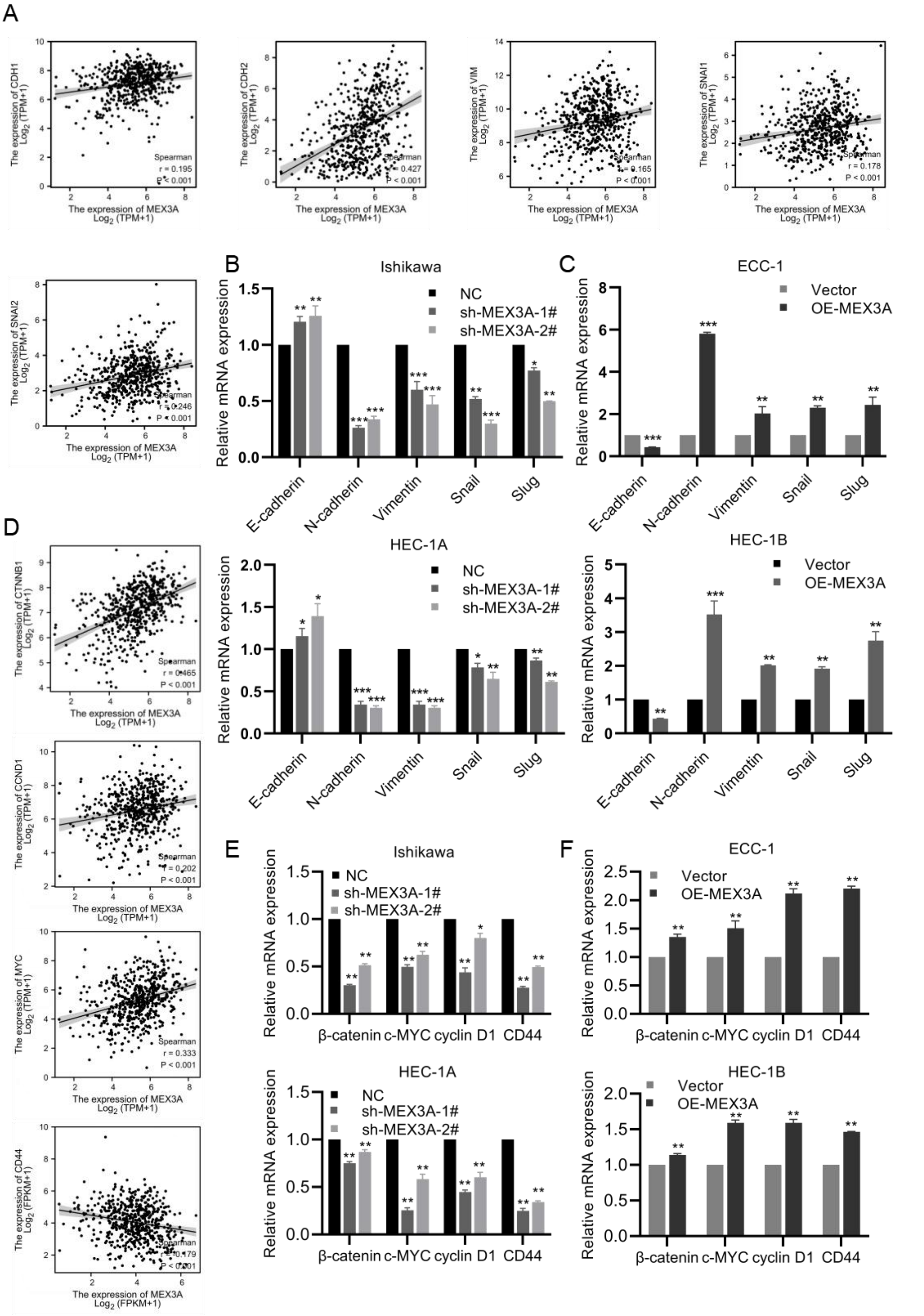

Supplementary Figure S3. MEX3A activates the EMT and Wnt/β-catenin signaling pathway. (A) Relationship between MEX3A and EMT-related genes (E-cadherin, N-cadherin, vimentin, Snail, and Slug) in TCGA. (B – C) The effect of MEX3A expression on EMT-related genes was investigated by qRT-PCR in MEX3A-knockdown and -overexpressing cells. (D) Relationship between MEX3A and Wnt/β-catenin signaling pathway-related genes (β-catenin, c-Myc, cyclin D1, and CD44) in TCGA. (E–F) The effect of MEX3A expression on the downstream target genes of β-catenin was measured by qRT-PCR in MEX3A-knockdown or -overexpression cells. *$P < 0.05$, **$P < 0.01$, ***$P < 0.001$.

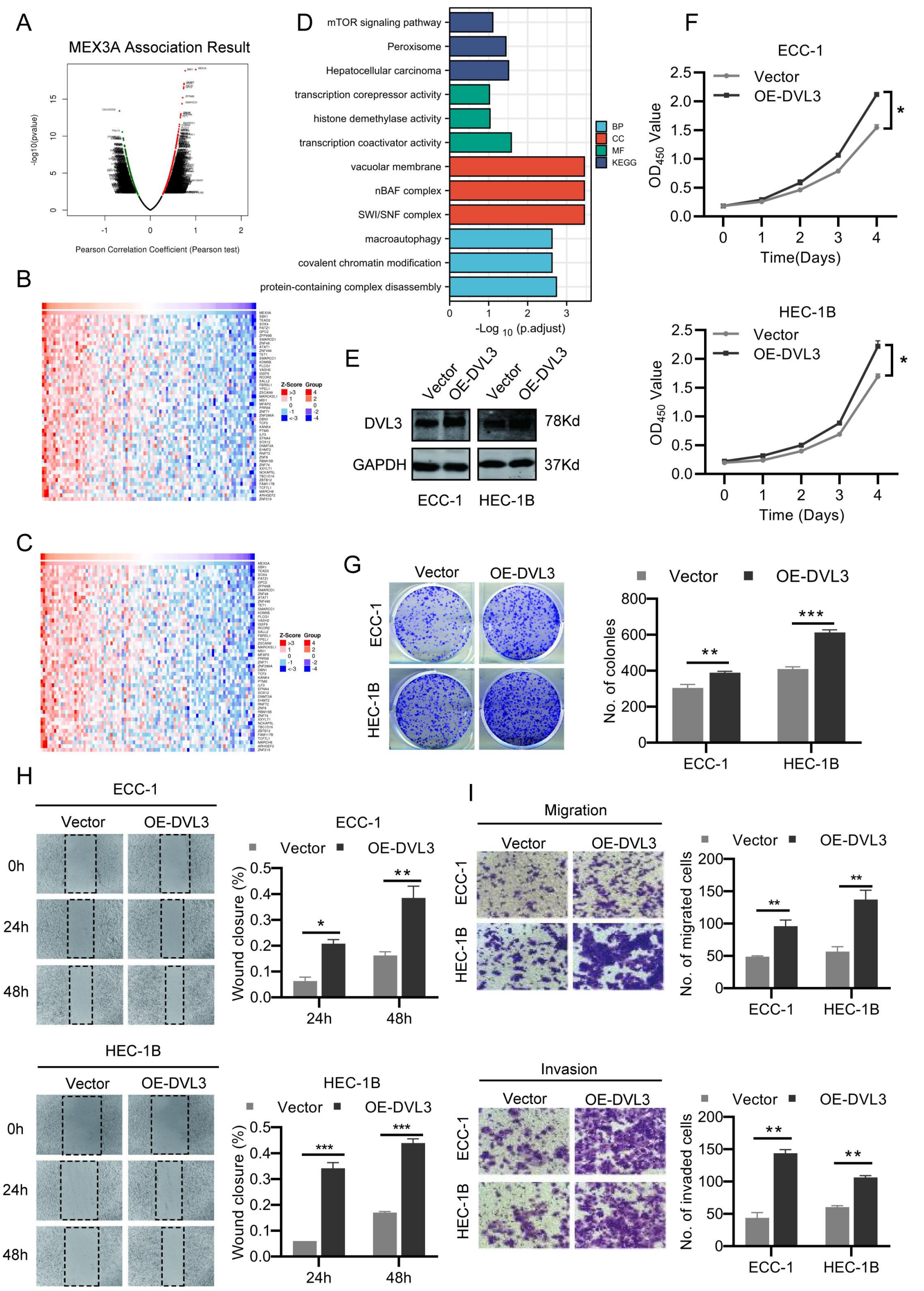

Supplementary Figure S4. MEX3A enhances the Wnt/β-catenin signaling pathway via DVL3. (A–C) Volcano plot and heatmap demonstrating the differentially-expressed genes based on MEX3A expression. (D) Functional enrichment analysis showing the significant pathways based on MEX3A expression. (E) The efficacy of DVL3 overexpression was detected using western blots. (F–G) The proliferation of ECC-1 and HEC-1B cells after DVL3 overexpression was measured using CCK-8 and colony formation assays. (H–I) The transwell and wound healing assays detected the migration and invasion abilities of DVL3-overexpressing cells. $*P < 0.05$, $**P < 0.01$, $***P < 0.001$.

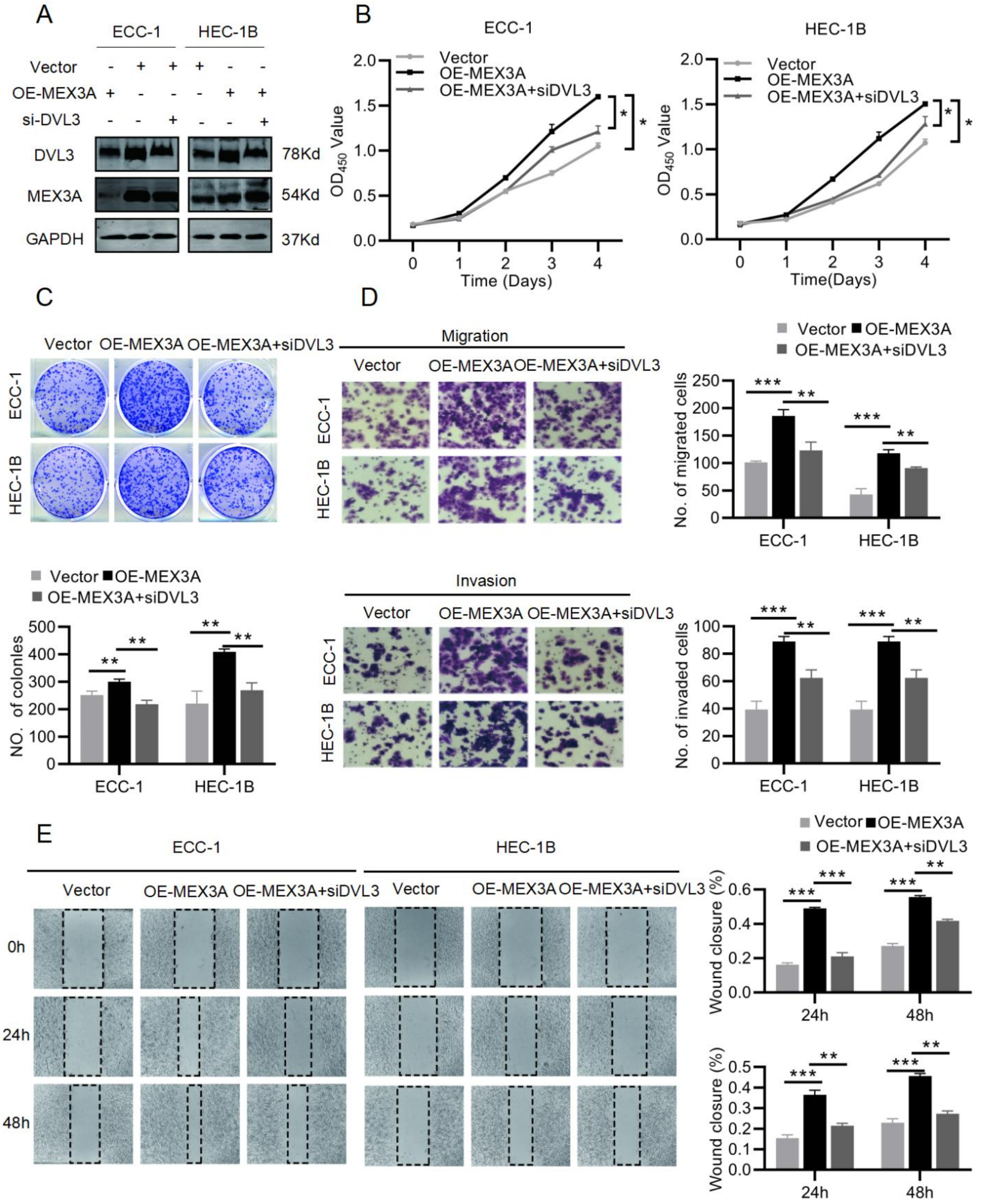

Supplementary Figure S5. DVL3 is vital for the function of MEX3A in EC cell proliferation and migration. (A) Protein levels of MEX3A and DVL3 in ECC-1 and HEC-1B cells after MEX3A overexpression or siDVL3 transfection. (B – C) CCK-8 and colony formation assays were performed in different treatment groups (Vector, OE-MEX3A, and OE-MEX3A+ si-DVL3). (D–E) Transwell and wound healing assays were performed using the treated ECC-1 and HEC-1B cells. *$P < 0.05$, **$P < 0.01$, ***$P < 0.001$.
